# Optoelectronic mixing with high-frequency graphene transistors

**DOI:** 10.1038/s41467-021-22943-1

**Published:** 2021-05-12

**Authors:** A. Montanaro, W. Wei, D. De Fazio, U. Sassi, G. Soavi, P. Aversa, A. C. Ferrari, H. Happy, P. Legagneux, E. Pallecchi

**Affiliations:** 1 Thales Research and Technology, Palaiseau, France; 2 Photonic Networks and Technologies Lab - CNIT, Pisa, Italy; 3 Univ. Lille, CNRS, Centrale Lille, Univ. Polyechnique Hauts-de-France, UMR8520 - IEMN Institut d’Electronique, de Microélectronique et de Nanotechnologie, Lille, France; 4 Cambridge Graphene Centre, University of Cambridge, Cambridge, UK; 5 LSI, CEA/DRF/lRAMIS, Ecole Polytechnique, CNRS, lnstitut Polytechnique de Paris, Palaiseau, France

**Keywords:** Optics and photonics, Optical properties and devices

## Abstract

Graphene is ideally suited for optoelectronics. It offers absorption at telecom wavelengths, high-frequency operation and CMOS-compatibility. We show how high speed optoelectronic mixing can be achieved with high frequency (~20 GHz bandwidth) graphene field effect transistors (GFETs). These devices mix an electrical signal injected into the GFET gate and a modulated optical signal onto a single layer graphene (SLG) channel. The photodetection mechanism and the resulting photocurrent sign depend on the SLG Fermi level (*E*_*F*_). At low *E*_*F*_ (<130 meV), a positive photocurrent is generated, while at large *E*_*F*_ (>130 meV), a negative photobolometric current appears. This allows our devices to operate up to at least 67 GHz. Our results pave the way for GFETs optoelectronic mixers for mm-wave applications, such as telecommunications and radio/light detection and ranging (RADAR/LIDARs.)

## Abstract (web summary)

Here, the authors report optoelectronic mixing up to 67 GHz using high-frequency back-gated graphene field effect transistors (GFETs). These devices mix an electrical signal injected into the GFET gate and a modulated optical signal onto a single layer graphene channel.

## Introduction

Mixers are a key component of modern communication modules^1^. In telecommunications and in radio detection and ranging (RADAR) systems^2^, the receiver analyzes the modulation of a carrier wave (or waveform) with frequencies in the microwave (3–30 GHz) or mm-wave (30–300 GHz) range, to extract information^3,4^. As signal processing is performed at near-zero frequencies (baseband^3,4^), frequency downconversion is required^3,4^. Downconversion is performed by mixing the modulated high-frequency signal centered around the radio frequency (RF) carrier frequency, *f*_RF_, with a local oscillator signal at frequency *f*_LO_. This translates the modulation centered around *f*_RF_ to *f*_IF_ = *f*_LO_ − *f*_RF_. The local oscillator frequency is typically set near *f*_RF_, so that *f*_IF_ is close to zero^3,4^. Superheterodyne receivers are a common type of radio receivers using frequency downconversion to process the original signal^5^. For multi-antenna systems, it is preferable to use a single optical signal as a local oscillator and distribute it to each antenna^6^, decreasing the receiver complexity and noise. For this purpose, one option is to use photodetectors (PDs) to transfer the local oscillator signal from the optical to the electrical domain^6^. After that, an electrical mixer is used^5^. A second option is to employ optoelectronic mixers (OEMs)^7^, i.e., PDs capable of mixing optical local oscillator with an electrical signal^7^. OEMs are particularly convenient in RADAR and light detection and ranging (LIDAR) applications^7–10^. State-of-the-art OEMs at 1.55 μm are based on III–V semiconductors epitaxially grown on InP^11,12^. These are efficient, but expensive, and can only be heterogeneously integrated in an Si platform^11,12^. Low cost and complementary metal-oxide-semiconductor (CMOS) compatible OEMs require CMOS-compatible materials absorbing light at 1.55 μm^13^.

Graphene is promising for optoelectronics^14–18^, with mobilities up to ~150,000 cm^2^ V^−1^ s^−1^ at room temperature (RT)^19^, a short (~1 ps) photocarrier lifetime^20–22^, and a 2.3% broadband light absorption (including telecom wavelengths)^23^. Graphene-based optoelectronic devices are compatible with Si platforms^16,24–27^. Therefore, graphene-based OEMs could combine telecom operation and CMOS compatibility.

Low frequency (2MHz) optoelectronic mixing in single-layer graphene (SLG) was studied in ref. ^28^ using a transistor structure with an on-chip bias resistor. This reported upconversion of a 2MHz signal, and downconversion of a 0.45 MHz one, in two types of OEMs consisting of a SLG field-effect transistor (GFET) and a bias resistor. For the first, the oscillating electrical signal was applied to the GFET drain (the optical signal illuminated the GFET channel), whereas in the second the signal was applied to the gate, and the mixing was proportional to the on-chip resistance. A 30 GHz bandwidth (BW) OEM based on SLG was reported in ref. ^29^, based on an SLG coplanar waveguide (GCPW) integrating a SLG channel grown by chemical vapor deposition (CVD). The RF signal was injected into the GCPW, whereas a 1.55 μm laser illuminated the channel. Optoelectronic mixing was based on the linear dependence of the photocurrent on both optical incident power (*P*_opt_) and voltage drop (*V*_bias_) along the channel. As the photocurrent is proportional to *P*_opt_*V*_bias_^30^, upconverted and downconverted signals were generated. This GCPW operated up to 30 GHz, with a conversion efficiency, (i.e., ratio of output power at *f*_IF_ and input power at *f*_RF_), of −85 dB for a 10 GHz modulated signal. The results in ref. ^29^ are far from state-of-the-art OEM performances achieved with III–V semiconductor-based uni-traveling carrier photodiodes: −22 dB conversion efficiency at 35 GHz^31^, and −40 dB at 100 GHz^12^. However, the CMOS integration of III–V semiconductors is challenging^13^. SLG is CMOS-compatible^16^ but, to technologically bridge the gap with III–V-based OEMs, BW, and conversion efficiency need to be improved^29^. Furthermore, the OEM in ref. ^29^ was a two-contact device operating only in the photoconductive regime, at a fixed Fermi level (*E*_*F*_).

Here, we present a 67 GHz GFET-OEM (three-contact device), exploiting the modulation of *E*_*F*_, that controls the photoconductivity. At low (equilibrium) *E*_*F*_ (<130 meV) the laser power induces interband transitions^32^, thus the charge carrier density, *n*, and the channel conductance increase (positive photoconductivity). At high *E*_*F*_ (>130 meV), the laser heating induces intraband transitions^32^. In this case, the hot carrier distribution reduces the effectiveness of the electronic screening^32^ which, in turn, leads to a higher scattering rate (e.g., owing to Coulomb impurities^32^ or strain disorder^33^). The increased scattering decreases the carrier mobility^32^ and the resulting photoconductivity is negative^32,34–36^. In our OEMs, an intensity-modulated (up to 67 GHz) laser beam illuminates part of the SLG channel, generating an AC photocurrent, proportional to the product of optical power and photoresponsivity. An RF signal applied to the gate of 20 GHz-BW GFETs modulates the photoresponsivity, mixing optical and electrical signals. The performance far exceeds that in ref. ^29^. The conversion efficiency (−67 dB) for a 67 GHz modulated optical signal (*f*_opt_) is 21 dB higher than ref. ^29^ at *f*_opt_ = 10 GHz, thanks to the use of an RF GFET with a strong coupling between the input RF signal and SLG (a 0.8 V signal induces *E*_*F*_ ~ 0.2 eV). Our results pave the way for the use of graphene in CMOS-compatible OEMs.

## Results

### Graphene growth and characterization

**Fig. 1:**
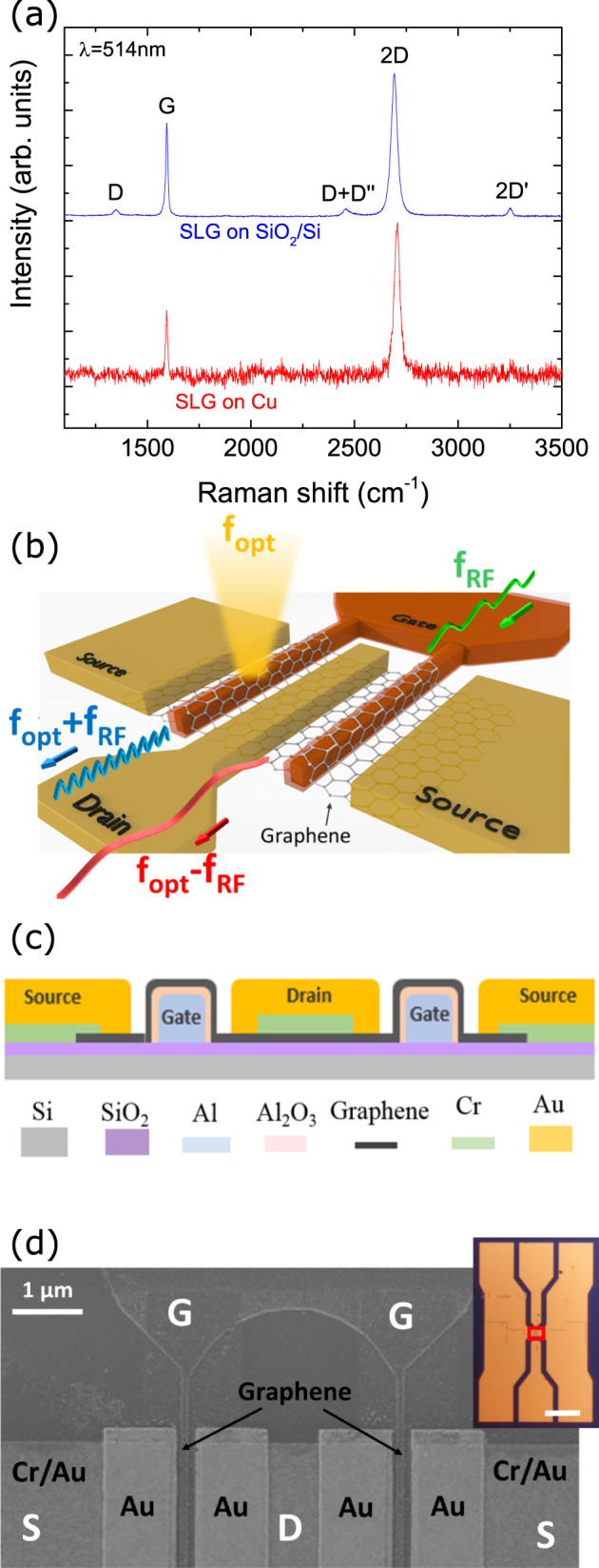
SLG Raman characterization and device description. **a** Representative Raman spectra at 514 nm of SLG as-grown on Cu (red), and after transfer on SiO_2_/Si (blue). **b** Principle of operation of our OEM. The mixing of the electrical signal at *f*_RF_ with the photodetected signal at *f*_opt_ generates two signals at the output (drain): *f*_opt_ + *f*_RF_ and *f*_opt_ − *f*_RF_. **c** Schematic GFET cross-section. The two Al gates (pale blue) are covered by a thin Al_2_O_3_ oxide (pink). SLG (black) is placed on the two gates. The drain and source contacts are Au (yellow) and Cr (green). **d** SEM image of GFET with dual-bottom gate finger covered by SLG. The metal in contact with SLG is Au. The inset shows the GFET (red rectangle) integrated into a coplanar waveguide (CPW) (scale bar: 100 μm).

SLG is grown via CVD on 35 μm-thick Cu foil, following ref. ^37^. The temperature, *T*, is raised to 1000 ^∘^C in an H_2_ atmosphere (~200 mTorr), and kept constant for 30 mins. In all, 5 sccm CH_4_ are then added to the 20 sccm H_2_ flow to start growth, for additional 30 mins at 300 mTorr. The sample is then cooled at ~1 mTorr to RT. We use Raman spectroscopy at 514nm to characterize the material. Figure 1a shows the Raman spectrum on Cu (red line), after Cu photoluminescence removal^38^. The D peak is absent, indicating negligible defects^39,40^. The 2D peak at ~2705 cm^−1^ is a single Lorentzian with full width at half maximum (FWHM) ~31 cm^−1^, a fingerprint of SLG^41^. The position of the G peak, Pos(G), is ~1593 cm^−1^, with FWHM(G) ~12 cm^−1^. The 2D to G intensity and area ratios are I(2D)/I(G) ~ 2.4, A(2D)/A(G) ~ 6.3.

To prevent ohmic losses at microwave frequencies, a high resistivity Si wafer (> 8000Ωcm) covered with  285nm SiO_2_ is used. SLG is wet transferred^42,43^ on it as follows. A poly(methyl methacrylate) (PMMA) layer is spin-coated on the surface of SLG/Cu and then placed in a solution of ammonium persulfate (APS) and deionized (DI) water for Cu etching^42^. The PMMA membrane with attached SLG is then immersed into a beaker filled with DI water for cleaning APS residuals. After, the PMMA/SLG stack is transferred onto the target substrate and the PMMA layer is removed. SLG is then ion etched to define the channel.

We then characterize via Raman spectroscopy the transferred SLG (blue curve, Fig. 1a). Both measurements on Cu and Si+SiO_2_ are performed under the same conditions of laser power, objective, wavelength, and accumulation time. The Raman signal of SLG on Cu is more noisy than on Si+SiO_2_ due to interference enhancement by the 300 nm SiO_2_ layer on Si^44,45^. For SLG on Si+SiO_2_ we have Pos(G) ~1594 cm^−1^, FWHM(G) ~11, Pos(2D) ~2691 cm^−1^, FWHM(2D) ~34 cm^−1^, I(2D)/I(G) ~1.6, A(2D)/A(G) ~4.5. This indicates p-doping ~300 meV^46,47^. I(D)/I(G) ~0.09 corresponds to a defect density ~4 × 10^10^ cm^−2^^40,48^, consistent with what is commonly observed in CVD-SLG^49^. It is possible to improve the process to get a smaller D peak^50^.

### Operational principle and device fabrication

Figure 1b is a sketch of our SLG OEM and illustrates its operational principle. It consists of a GFET with a symmetric dual-bottom gate finger. This layout is commonly used for RF applications^51^ and GFETs^52^, and is well suited for GCPWs^53^. Dual-gate finger FETs have a more compact design and a reduced small-signal gate resistance compared with single-gate configurations, for a given equivalent channel width, resulting in a higher voltage gain^54^. A laser beam is modulated at *f*_opt_ and focused on the GFET channel. As a result, a photocurrent that contains an AC component at *f*_opt_ flows through the SLG channel. If a RF signal *f*_RF_ is applied to the gate, the output current presents a term at *f*_RF_. When both optical and electric signals are applied, the device acts as an OEM: the output contains the product of the two signals, and two AC components at *f*_opt_ + *f*_RF_ and *f*_opt_ − *f*_RF_ appear.

A schematic cross-section of the bottom gate GFET is in Fig. 1c. The fabrication starts by patterning the dual-bottom gate finger by e-beam lithography (EBPG 5000 Plus). The gates are made of a 40 nm-thick Al layer deposited by evaporation. A 4 nm Al_2_O_3_ layer is formed on top of the gates by exposing the substrate to pure oxygen for 30 mins^55^ with an Oxford Plasmalab80Plus at ~100 mTorr. This thin oxide acts as gate dielectric. The source and drain contacts are made in a two-steps process. First, Cr/Au (5/50 nm) pre-contacts are deposited on SLG. Then, ohmic contacts are obtained by placing 30 nm Au on the Cr/Au-SLG junction. Finally, a CPW is built with a Ni/Au film (50/300 nm). Figure 1d is a scanning electron microscopy (SEM) image of the bottom gates covered by SLG. The inset shows a GFET integrated into the CPW. The red square indicates the area occupied by the GFET. The bottom gate GFET design is suitable for OEMs since (1) the SLG channel is on the gate and can be directly illuminated; (2) the use of a thin (4 nm) Al_2_O_3_ dielectric and short gate (<0.4 μm or less) ensures high-frequency operation^5,56,57^.

The device has a cutoff frequency (not de-embedded) *f*_*t*_ ~ 25 GHz, and a maximum oscillating frequency *f*_max_ ~ 14GHz, as deduced from the S-parameters measured with a Vector Network Analyzer (VNA, Agilent, E8361A). To calibrate the VNA, we use the Line-Reflect-Reflect-Match approach^58^. This allows us to eliminate errors in S-measurements introduced by the environment, such as cables and probe tips used to contact the device under test, and the VNA non-idealities.

### Electrical and optoelectronic measurements

**Fig. 2:**
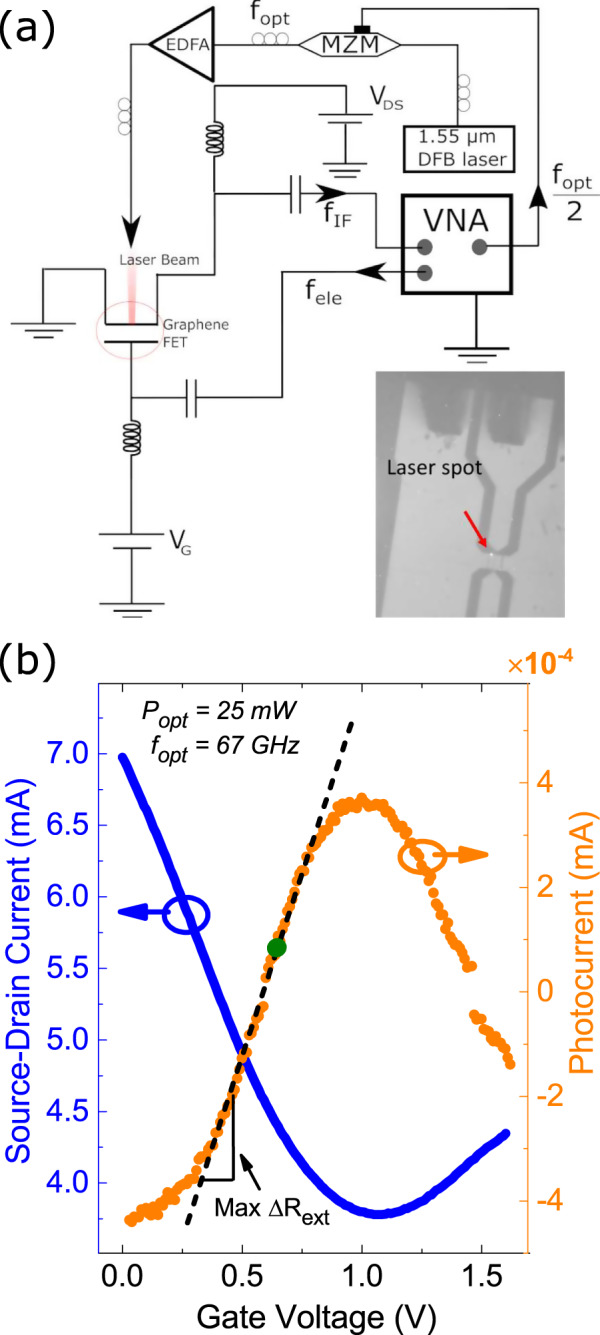
Measurement setup and device characteristics. **a** Experimental setup: a CW laser is modulated via a MZM. It is then amplified with an EDFA and focused on the GFET. An AC signal is applied to the gate. The output f_*IF*_ is measured on a VNA. Inset: optical image of device with laser focused on the channel. **b** Blue curve: source-drain current versus gate voltage, for *V*_DS_ = 200 mV. Orange curve: photocurrent versus gate voltage, generated by a 25 mW beam focused on the SLG channel.

The setup in Fig. 2a is used to measure photocurrent and optoelectronic mixing. The output of a 1.55 μm distributed feedback laser is modulated by a Mach Zehnder modulator in the double sideband suppression carrier mode^59^, to obtain a modulated beam at *f*_opt_. This is then amplified with an Erbium-doped fiber amplifier. The maximum *f*_opt_ that our setup can probe is 67 GHz. The diameter of the focused laser spot is ~2 μm (inset of Fig. 2a). The maximum power impinging on the sample is ~60 mW, which corresponds to ~20 mW/μm^2^. The gate and drain are connected to a VNA with two high-frequency (67 GHz) air coplanar probes. Bias tees are used to add a DC bias to channel and gate electrodes, and to measure the DC currents and voltages with a Source-Measure-Unit (Keithley 2636B). After illuminating the device, we verify the stability of the signal before measuring the RF photocurrent, whereas monitoring the DC value of the channel resistance, to ensure that no damage nor significant modification is induced by the laser power or DC bias. We do not observe any degradation or time-dependent drift in the DC or RF currents over a period of at least 3 h, the typical measurement time.

We now present the results for a representative OEM with SLG channel width *W* = 24 μm, length *L* = 400 nm, and gate length *L*_*G*_ = 200 nm. The blue curve in Fig. 2b is the source-drain current, *I*_DS_, as a function of gate voltage, *V*_GS_, at *V*_DS_ = 200 mV, which shows the typical ambipolar conduction behavior of a GFET (i.e, electrical conductivity due to electrons/holes (*e*/*h*), depending on the position of *E*_*F*_ with respect to the charge neutrality point, CNP)^60^. The minimum conductance is reached at *V*_GS_ = 1.1 V, which corresponds to the CNP voltage (*V*_CNP_). When *V*_GS_ increases (decreases) with respect to *V*_CNP_, the *e*(*h*) density increases, leading to a reduction of channel resistivity, so an increase of the current flowing in the channel^60^. *μ* is calculated as *μ* = *Lg*_*m*_/(W ⋅ C_*G*_V_DS_)^61^. The transconductance g_*m*_ = *dI*_DS_/*dV*_GS_^13^ is obtained from the transfer characteristic *I*_DS_(*V*_GS_) at V_DS_ = 10 mV. The gate capacitance is *C*_*ox*_ is ~5 *fF/*μm, obtained from S parameters measurements on 60 devices of the same kind^55^. We get *μ* ~ 2500 cm^2^ V^−1^ s^−1^, consistent with that of non-encapsulated CVD-SLG^37^.

We now consider the photoresponse. The OEM is biased at *V*_DS_ = 200 mV and illuminated with a laser modulated at *f*_opt_ = 67 GHz. The electrical power *P*_RF_ measured by the VNA is used to derive the photocurrent *I*_ph_. From Joule’s law^13^ ${I}_{\rm{ph}}=\sqrt{\frac{{P}_{\rm{RF}}}{{Z}_{\rm{VNA}}}}$, with *Z*_VNA_ = 50 Ω the VNA input impedance. The use of bias tees allows us to simultaneously measure the DC component, i.e., the dark current (blue curve in Fig. 2b) and the AC component, i.e., the photocurrent (orange curve in Fig. 2b) as a function of *V*_GS_, for a 25 mW incident optical power. The photocurrent, *I*_ph_, sign depends on V_GS_. *I*_ph_ is positive and has a local maximum close to the CNP. At low (equilibrium) *E*_*F*_ (<130 meV), the laser power induces interband heating^32^, thus an increase of *n* (positive photoconductivity). Therefore, the photocurrent has the same sign as the DC current in the channel, owing to the DC bias. At high *E*_*F*_ > 130 meV, the sign of the photocurrent is opposite to the DC current (negative photoconductivity). In this case, laser heating induces intraband transitions, which lead to a reduction of electronic screening of the long-range Coulomb interaction between SLG’s carriers and charged impurities in the substrate^22,32^. The *E*_*F*_ at which the transition between positive and negative photocurrent takes place is ~0.1–0.2 eV^32,34^. The value depends on the charge transport scattering rate in SLG, i.e., the mean time interval between two collisions in the diffusive transport picture^62^, and on the charge neutrality region width^63^ (see Methods). In our experiment, we observe this transition at ~130 meV.

The external photoresponsivity, *R*_ext_, is defined as^15,64^ *R*_ext_ = $\frac{| {I}_{\rm{ph}}| }{{P}_{\rm{c}}{{\rm{w}}_{\rm{eff}}}}$, with ${P}_{\rm{c}}{{\rm{w}}_{\rm{eff}}}$ = 31%*P*_cw_ the fraction of the optical power coupled to the SLG channel. We get *R*_ext_ ~ 0.22 mA/W. For *V*_GS_ = 0V, the device reaches its maximum *I*_ph_ ~ −4.2 × 10^−4^ mA and the photocurrent generated by a 67 GHz laser modulation is measured as a function of DC bias and optical power.

**Fig. 3:**
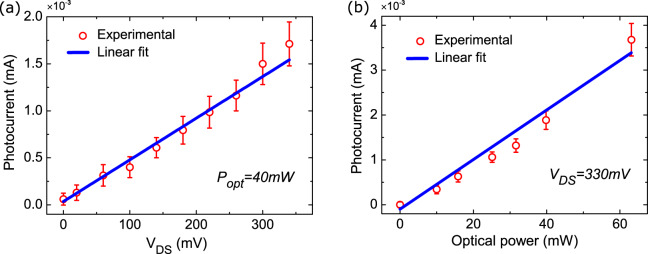
Photocurrent measurements. **a** Photocurrent as a function of *V*_DS_ at 40 mW optical power, **b** photocurrent as a function of the optical power, at *V*_DS_ = 330 mV. Error bars are obtained from the VNA measurement noise standard deviation.

**Fig. 4:**
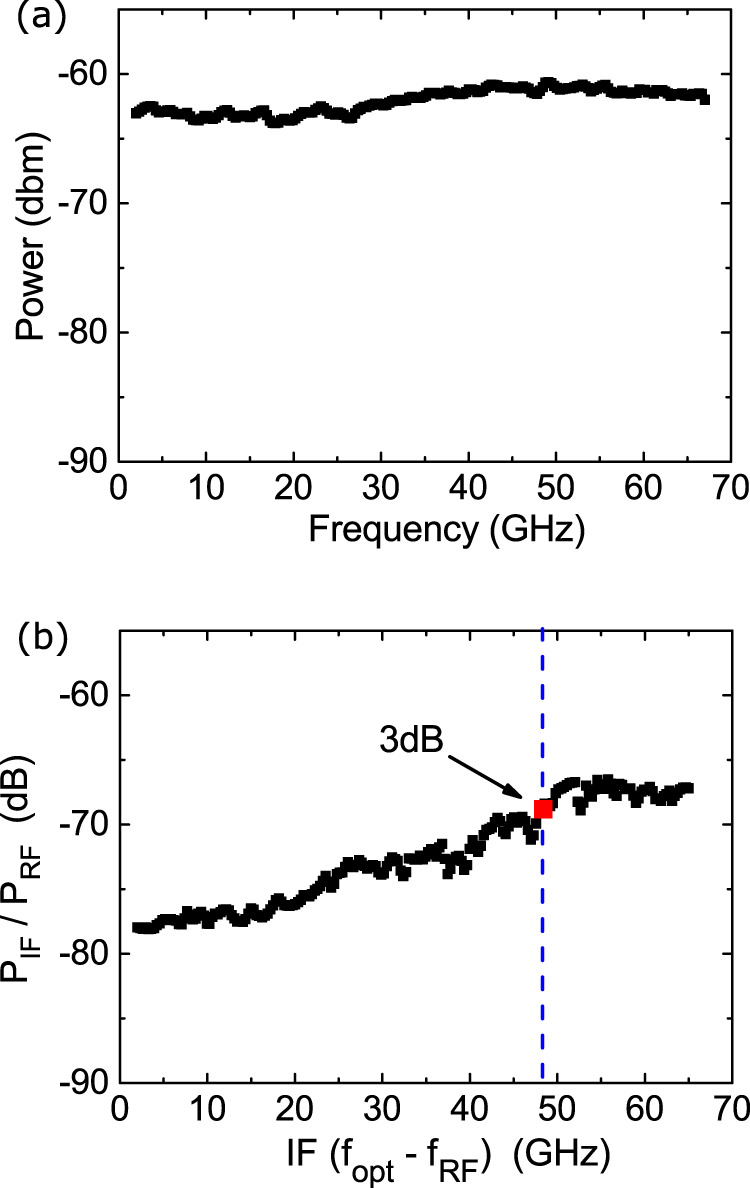
RF Optoelectronic characterization. **a** Maximum photodetected power at *V*_GS_ = 0V, *V*_DS_ = 330 mV, as a function of *f*_opt_. **b** *P*_IF_/*P*_RF_ at *V*_GS_ = 0.6V, *V*_DS_ = 330 mV. Optical power in **a**, **b** is 60 mW.

Figures 3a, b plot the photocurrent as a function of DC bias at *P*_opt_ = 40 mW and as a function of the optical power for *V*_DS_ = 330 mV. The response is linear in both cases, as expected for a photoconductor^34^. The frequency response of the photodetected power is then measured as a function of *f*_opt_, Fig. 4a. We get a flat response over the whole band that can be investigated by our VNA, showing that the intrinsic photodetection BW is >67 GHz. The photocurrent can originate from the SLG channel located above the gate, or from the SLG-metal contacts that are not gated (see Fig. 1c). As the photoresponse strongly depends on V_GS_, as shown in Fig. 2b, we ascribe it to the illuminated part of the SLG channel located above the gate. If both contacts are illuminated, the photocurrents are opposite and cancel out. If the photocurrent originates mainly from one SLG-metal contact, the photocurrent at *V*_DS_ = 0V should be significant. *R*_ext_ ~ 0.8 mA/W was reported in ref. ^65^ for a detector exploiting the metal-SLG contacts, at *V*_DS_ = 0V. As seen in Fig. 3, at *V*_*DS*_ = 0V the photocurrent is negligible. We also rule out possible asymmetric heating effects on the channel, owing to beam location, by performing a photocurrent measurement as a function of laser spot position. We observe a very weak response at *V*_DS_ = 0V, regardless of laser spot position, as shown in Methods, Fig. 10. Thus, the role of contacts can be neglected.

In order to operate the device as an OEM (instead of a PD), an RF signal *f*_RF_ is added to the DC gate, Fig. 1b. *f*_opt_ is maintained at 67 GHz, whereas *f*_RF_ is swept between 2 and 65 GHz. A VNA is used to record *P*_IF_ and the transistor power at the intermediate frequency *f*_IF_ = *f*_opt_−*f*_RF_.

An important parameter for OEMs is the downconversion efficiency^5^: *P*_IF_/*P*_RF_, with *P*_RF_ the power at the source and *P*_IF_ that measured at the VNA. For our device, the maximum *P*_IF_/*P*_RF_ is −67 dB at *V*_GS_ = 0.6 V. For this *V*_GS_, Fig. 4b plots *P*_IF_/*P*_RF_ as a function of *f*_IF_. The BW and *P*_IF_/*P*_RF_ of our OEMs far exceed (+37 GHz in BW and 2 orders of magnitude in *P*_IF_/*P*_RF_) those of ref. ^29^, where the input RF signal modulates the SLG bias (resistive coupling), thus the photocurrent amplitude. In our OEMs, the input RF signal is coupled to SLG via the gate oxide (capacitive coupling), which results in a modulation of *E*_*F*_ and, consequently, in a change of the photocurrent mechanism and sign. These high BW and *P*_IF_/*P*_RF_ come from the strong coupling (i.e., strong electric field for a small applied voltage, 0.25 V/nm) between the input RF signal (applied to the Al back-gate) and the SLG channel, thanks to the use of a ~4 nm oxide. As a consequence, an efficient field effect is achieved^60^. A signal with an amplitude ~0.8 V induces a *E*_*F*_ modulation ~0.2 eV. Thus, a small signal (0.8 V) is needed to obtain optoelectronic mixing. The high-frequency operation of the GFET (~20 GHz, 3 dB BW, Fig. 4b) comes from the short channel length (400 nm) and the small gate capacitance *C*_ox_ ~ 60 fF^66^.

**Fig. 5:**
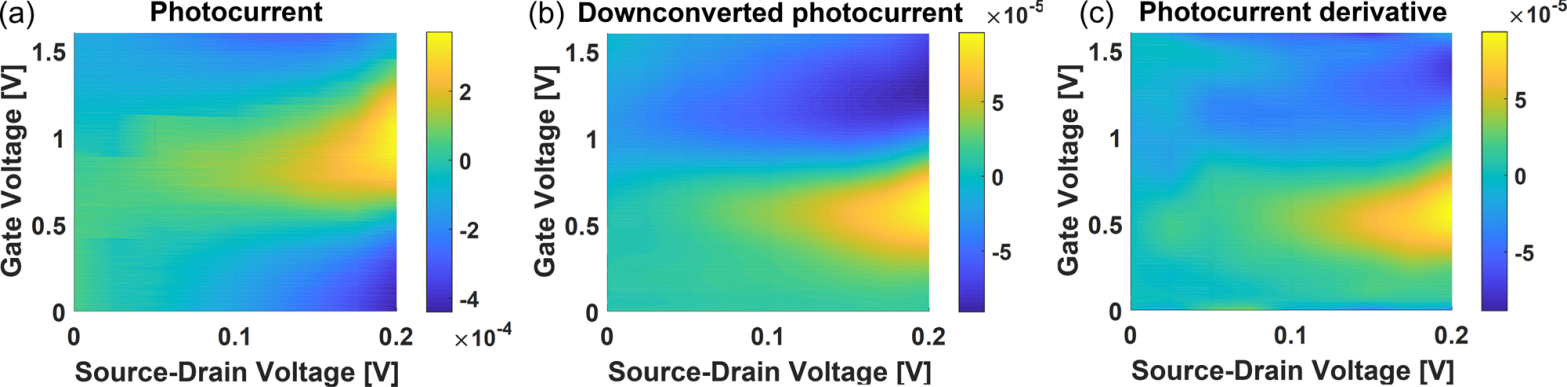
*V*_GS_–*V*_DS_ maps. **a** Photocurrent map as a function of *V*_GS_, *V*_DS_. **b** Downconverted photocurrent map as a function of *V*_GS_, *V*_DS_. **c** Derivative of **a** with respect to *V*_GS_. The photocurrent values are in mA.

**Fig. 6:**
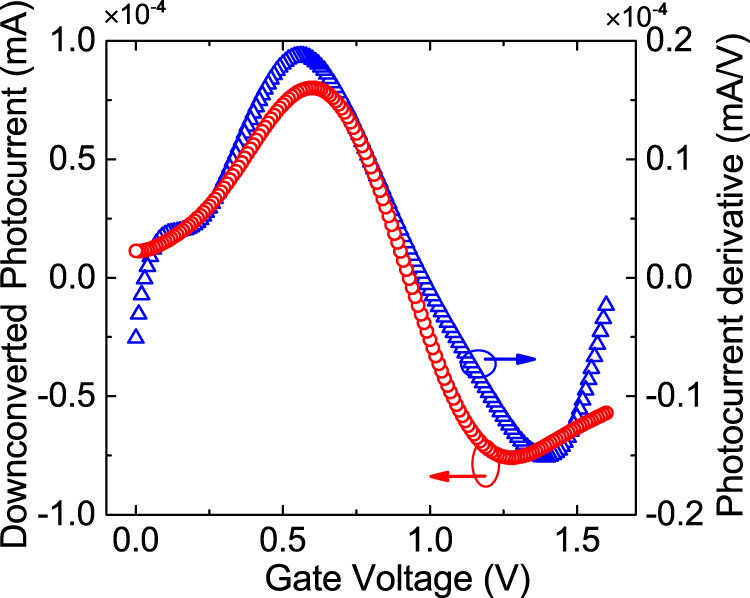
Downconversion efficiency vs *V*_*GS*_. Red curve: cut of Fig. 5c for *V*_DS_ = 200 mV. Blue curve: cut of Fig. 5b for *V*_DS_ = 200 mV.

Figure 5a is a color map of the 67 GHz photocurrent as a function of *V*_GS_, *V*_DS_. We then add to the DC gate bias an electrical signal at 10 GHz. The resulting downconverted photocurrent at *f*_IF_ = 57 GHz is plotted as a function of *V*_DS_, *V*_GS_ in Fig. 5b. By differentiating the map in Fig. 5a with respect to *V*_GS_, we obtain Fig. 5c, which resembles Fig. 5b. This is best seen in Fig. 6, which plots both values as a function of *V*_GS_ for *V*_DS_ = 200 mV. The curves of the downconverted photocurrent and of the derivative of the photocurrent can be superposed. This result is valid regardless of frequency, see Methods Fig. 9.

## Discussion

This behavior can be explained by a small-signal analysis.

Let us consider the modulated optical power impinging on the PD, *P*_opt_ = *P*_cw_ + *P*_mod_*sin*(2*πf*_opt_*t*), with *P*_mod_ the amplitude of the varying part of the optical power. The photocurrent is proportional to *P*_opt_ through the factor *R*_ext_. This depends on *V*_GS_, as for Fig. 2b, and is almost independent on *f*_opt_, Fig. 4a. Therefore, the photocurrent can be written as:

1 $${I}_{\rm{ph}}({V}_{\rm{GS}})={R}_{\rm{ext}}({V}_{\rm{GS}})[{P}_{\rm{cw}}+{P}_{\rm{mod}}sin(2\pi {f}_{\rm{opt}}t)]$$

By applying to the gate a DC bias ${\overline{V}}_{\rm{GS}}$ and a small signal ${\delta }_{{V}_{\rm{GS}}}sin(2\pi {f}_{\rm{RF}}t)$, we get:

2 $${R}_{\rm{ext}}({V}_{\rm{GS}})={R}_{{{\rm{ext}}}_{\rm{DC}}}({\overline{V}}_{\rm{GS}})+{\delta }_{{V}_{\rm{GS}}}{{{\Delta }}}_{{R}_{\rm{ext}}}sin(2\pi {f}_{\rm{RF}}t)$$

where

3 $${{{\Delta }}}_{{R}_{\rm{ext}}}=\beta ({f}_{\rm{RF}})\frac{d{R}_{\rm{ext}}({V}_{\rm{GS}})}{d{V}_{\rm{GS}}}{| }_{{V}_{\rm{GS}} = {\overline{V}}_{\rm{GS}}}$$

We include dependence on injected electrical frequency through a frequency-dependent proportionality constant *β*(*f*_RF_). The total photocurrent has four terms:

4 $${I}_{\rm{ph}}	= {R}_{{{\rm{ext}}}_{\rm{DC}}}({\overline{V}}_{\rm{GS}}){P}_{\rm{cw}}+{\delta }_{{V}_{\rm{GS}}}{{{\Delta }}}_{{R}_{\rm{ext}}}{P}_{\rm{cw}}sin(2\pi {f}_{\rm{RF}}t)\\ 	\quad +\,{R}_{{{\rm{ext}}}_{\rm{DC}}}({\overline{V}}_{\rm{GS}}){P}_{\rm{mod}}sin(2\pi {f}_{\rm{opt}}t)\\ 	\quad + \,{\delta }_{{V}_{\rm{GS}}}{{{\Delta }}}_{{R}_{\rm{ext}}}{P}_{\rm{mod}}sin(2\pi {f}_{\rm{RF}}t)sin(2\pi {f}_{\rm{opt}}t)$$

The first is the DC photocurrent. The second describes the DC photocurrent modulated by the electrical signal. The third represents the photocurrent modulated at *f*_opt_, Fig. 2b. The fourth describes the optoelectronic mixing and can be rewritten as:

5 $${\delta }_{{V}_{\rm{GS}}}{{{\Delta }}}_{{R}_{\rm{ext}}}{P}_{\rm{mod}}\,sin(2\pi {f}_{\rm{RF}}t)\,sin(2\pi {f}_{\rm{opt}}t)	= \frac{1}{2}{\delta }_{{V}_{\rm{GS}}}{{{\Delta }}}_{{R}_{\rm{ext}}}{P}_{\rm{mod}}\left\{cos[2\pi ({f}_{\rm{RF}}-{f}_{\rm{opt}})t]\right.\\ 	\quad +\left.- \,cos[2\pi ({f}_{\rm{RF}}+{f}_{\rm{opt}})t)\right\}$$

Equation (5) has two components at *f*_opt_ + *f*_RF_ and *f*_IF_ = *f*_opt_ − *f*_RF_. It shows that the mixed signal depends exclusively on ${{{\Delta }}}_{{R}_{\rm{ext}}}$, i.e., on the derivative of *R*_ext_ with respect to *V*_GS_, not on *R*_ext_ itself, in accordance with Fig. 5. ${{{\Delta }}}_{{R}_{\rm{ext}}}$ is maximum for *V*_GS_ ~ 0.6 V, Fig. 6, i.e., in a region where the photocurrent changes sign, indicated in Fig. 2b with a green dot. The sharper is the transition between the two competing phenomena (photoconductive and bolometric) generating the photocurrent, the higher is ${{{\Delta }}}_{{R}_{\rm{ext}}}$ and the optoelectronic-mixing efficiency.

**Fig. 7:**
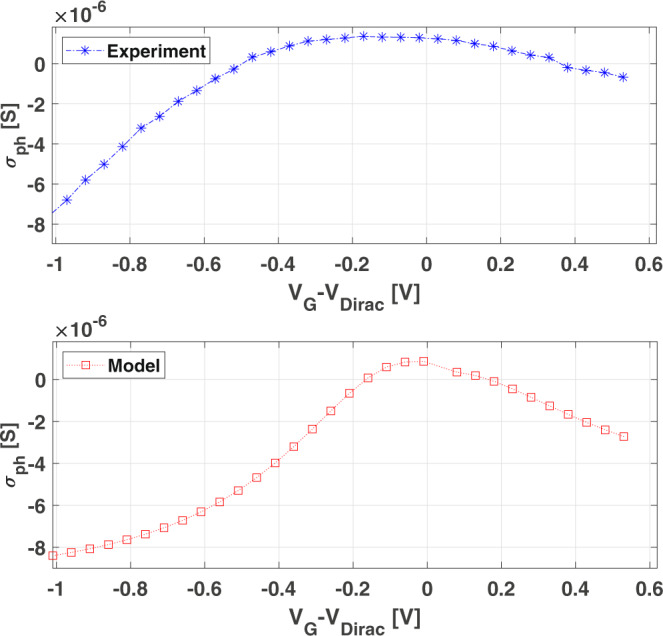
Comparison between measured and calculated photoconductivity. **a** Measured and **b** calculated photoconductivity of illuminated SLG channel.

Since R_ext_ is proportional to the photoconductivity *σ*_ph_(*V*_GS_)^30^, the optimization of the mixing efficiency (${{{\Delta }}}_{{R}_{\rm{ext}}}$) requires $\frac{d{\sigma }_{\rm{ph}}}{d{V}_{\rm{GS}}}$ to be maximized. *σ*_ph_(*V*_GS_) depends on several factors, such as the residual charge carrier density, *n*_0_^63^, and the dominating scattering mechanisms^63,67^. So, it depends on SLG quality and its dielectric environment. We compute *σ*_ph_(*V*_GS_) from the Drude model for free carrier conductivity in SLG^63^:

6 $$\sigma ({T}_{e},{\mu }_{c})=\frac{D({T}_{e},{\mu }_{c})}{\pi {{\Gamma }}({T}_{e},{\mu }_{c})}$$

where *T*_*e*_ is the electron temperature, Γ is the scattering rate, *D* is the Drude weight^63,67^, and *μ*_*c*_ is the chemical potential. We calculate *μ*_*c*_ (see Methods for details), and then the effect of *e*-*h* puddles by replacing *μ*_*c*_ with ${\mu }_{c}\to \root4\of{{\mu }_{c}^{4}+{{\Delta }}{{\mu }_{\rm{puddles}}}^{4}}$^63^, where ${{{\Delta }}}_{{\mu }_{\rm{puddles}}}$ is the energy width of the puddles region^63^. The photoconductivity due to illumination of the SLG channel at a given *μ*_*c*_ is then given by^63^:

7 $${\sigma }_{\rm{ph}}=\sigma ({T}_{\rm{light}})-\sigma ({T}_{\rm{dark}})=\frac{D({T}_{\rm{light}})}{\pi {{\Gamma }}({T}_{\rm{light}})}-\frac{D({T}_{\rm{dark}})}{\pi {{\Gamma }}({T}_{\rm{dark}})}$$

where *T*_light_ is the hot-electron temperature of the illuminated SLG and *T*_dark_ is the electron temperature in dark, see Methods for details. Figure 7 plots the measured photoconductivity (blue) and the computed one (red) using Eq. (7). We calculate Δ*μ*_puddles_ from *n*_0_, extracted from the experimental DC measurement of the conductivity (see Methods). We calculate Γ from the measured *μ* (see Methods). We get Γ ~ 50 meV, which corresponds to a scattering time *τ* ~ 80 fs at E_*F*_ ~ 200 meV, at the maximum *V*_GS_, in agreement with experiments.

We then calculate $\frac{d{\sigma }_{\rm{ph}}}{d{V}_{\rm{GS}}}$ to find the maximum downconversion efficiency. This can be increased by ~27 dB (power unit) for SLG with *μ*_fe_ ~30,000 cm^2^ V^−1^ s^−1^ (*τ* = 1/Γ ~ 0.6 ps) and *n*_0_ ~ 10^11^ cm^−2^, see Methods. Thus, the control of the transition between the two different photocurrent mechanisms could lead to a maximization of ${{{\Delta }}}_{{R}_{\rm{ext}}}$ and, in turn, a maximization of downconversion efficiency.

The 3 dB BW is ~19.7 GHz when operated as OEM, Fig. 4. This behavior is modeled in Eq. (3) by including the factor *β*(*f*_RF_). To understand the optoelectronic-mixing dependence on *f*_RF_ and, thus, on *f*_IF_ (*f*_opt_ being fixed), we consider a typical figure of merit of high-frequency transistors: the transducer power gain^5^, defined as: G_*T*_ = $\frac{{P}_{\rm{load}}}{{P}_{\rm{avs}}}$, where *P*_load_ is the power delivered to the load and *P*_avs_ is the source power. *G*_*T*_ coincides with the modulus of the S_21_ parameter when source and load are matched^5^. This is the case in our measurements, where the power is delivered from the VNA 50Ω-source and measured on a 50Ω receiver. *G*_*T*_ is close to the S_21_ parameters. An external impedance matching could increase the downconversion efficiency by maximizing the power delivered by the GFET, other than increasing BW. We do not observe saturation in the photodetected signal at the highest optical power available in our setup (60 mW). Thus, illuminating a wider channel surface, while maintaining the same optical power density, should increase the downconversion efficiency. An enhancement of SLG-light interaction can also improve the downconversion efficiency. In all, ~70% absorption could be achieved by integrating SLG on a waveguide^68^. This could enhance the downconversion efficiency by ~30 dB, compared with the normal incidence case (~2.3% absorption^23^).

In summary, we reported high-frequency graphene transistors operating as OEMs for frequencies up to at least 67 GHz. The photodetection BW exceeds 67 GHz. The BW of the devices operated as OEMs is 19.7 GHz. The conversion efficiency is at least two orders of magnitude higher than previous graphene OEMs^29^. It can be further increased using high-quality samples with *μ* ~ 10,000–100,000 cm^2^ V^−1^ s^−1^ and increasing light-matter interaction. This can increase the downconversion efficiency >50 dB, overcoming state-of the-art performances of OEMs based on any other technology^31^. Our frequency operation is already comparable with state-of-the-art performance of OEMs based on any other technology^31^. Thus, our work paves the way for the use of graphene-based transistors as OEMs in applications exploiting mm-waves, such as telecommunications and RADAR/LIDAR.

## Methods

### Photocurrent modeling

Equations 1–5 show that the optoelectronic-mixing efficiency is proportional to ${{{\Delta }}}_{{R}_{\rm{ext}}}$, which can be expressed as:

8 $${{{\Delta }}}_{{R}_{\rm{ext}}}=\beta ({f}_{\rm{RF}})\frac{d{R}_{\rm{ext}}({V}_{\rm{GS}})}{d{V}_{\rm{GS}}}{| }_{{V}_{\rm{GS}} = {\overline{V}}_{\rm{GS}}}$$

Since R_ext_ is proportional to *σ*_ph_(*V*_GS_)^30^, the optimization of the mixing efficiency requires $\frac{d{\sigma }_{\rm{ph}}}{d{V}_{\rm{GS}}}$ to be maximized. The mixing efficiency can be related via ${{{\Delta }}}_{{R}_{\rm{ext}}}$ to SLG’s *μ* and *n*^63,67^. The Drude model for free carrier conductivity in SLG gives^63^:

9 $$\sigma (\omega ,{T}_{e})=\frac{D({T}_{e})}{\pi [{{\Gamma }}({T}_{e})-i\omega ]}$$

In the case of Dirac Fermions the Drude weight is^63^:

10 $$D({T}_{e})=\frac{2{e}^{2}}{{\hslash }^{2}}{k}_{B}{T}_{e}ln\left[2cosh\left(\frac{{\mu }_{c}({T}_{e})}{2{k}_{B}{T}_{e}}\right)\right]$$

with *k*_*B*_ the Boltzmann constant. In the GHz range, *ω* ~ 10^9^−10^10^ rad/s. This value is negligible compared to our Γ, which lies in the range 10^12^−10^13^ rad/s^32^. The photoconductivity owing to the illumination of the SLG channel is then ^63^:

11 $${\sigma }_{\rm{ph}}=\sigma ({T}_{\rm{light}})-\sigma ({T}_{\rm{dark}})=\frac{D({T}_{\rm{light}})}{\pi {{\Gamma }}({T}_{\rm{light}})}-\frac{D({T}_{\rm{dark}})}{\pi {{\Gamma }}({T}_{\rm{dark}})}$$

This depends on *T*, as experimentally shown in ref. ^34^. Increasing *T* decreases the photodetection efficiency^34^. This is consistent with the screening reduction of the scattering mechanism while increasing T_*e*_^32^, since a smaller *T* change between dark and illumination conditions takes place.

*μ*_*c*_ is *T*-dependent. It decreases while increasing *T* to keep the number of conduction band carriers constant^69^. So, it is lower in illumination conditions with respect to dark. To account for this, we compute *μ*_*c*_ by numerical inversion of the following *T*-dependent formula^60,63^:

12 $$\frac{2}{\pi }\frac{{({k}_{B}T)}^{2}}{{(\hslash {v}_{0})}^{2}}\left[L{i}_{2}\left(-{e}^{\frac{-{\mu }_{c}}{{k}_{B}T}}\right)-L{i}_{2}\left(-{e}^{\frac{{\mu }_{c}}{{k}_{B}T}}\right)\right]=\frac{{C}_{\rm{ox}}{V}_{\rm{GS}}}{e}-\frac{{\mu }_{c}{C}_{\rm{ox}}}{{e}^{2}}$$

In Eq. (12), *C*_ox_ is the geometrical gate capacitance per unit area, *v*_0_ is the Fermi velocity and *Li*_2_ is the dilogarithm function. This is valid in low and high doping and also includes the effects of quantum capacitance. Γ is calculated from *μ*_*fe*_ ~ 2500 cm^2^/(Vs) using^16^:

13 $${{\Gamma }}=\frac{1}{\tau }=\frac{e{v}_{F}^{2}}{{\mu }_{c}({T}_{e}){\mu }_{\rm{fe}}}$$

We get Γ ~ 80 meV, i.e. *τ* ~ 50 fs for *μ*_*c*_ ~ 0.2 eV. Charge puddles are taken into account by replacing *μ*_*c*_ with^63^:

14 $${\mu }_{c}\to \root4\of{{\mu }_{c}^{4}+{{\Delta }}{{\mu }_{\rm{puddles}}}^{4}}$$

Δ*μ*_puddles_ is calculated as^60^:

15 $${{\Delta }}{\mu }_{\rm{puddles}}=\sqrt{{n}_{0}\pi }\hslash {v}_{F} \sim 120{\rm{meV}}$$

Here, *n*_0_ ~ 5 ⋅ 10^11^ cm^−2^ extracted from the measured DC minimum of conductivity *σ*_min_^60^:

16 $${n}_{0}=\frac{{\sigma }_{\rm{min}}}{e{\mu }_{\rm{fe}}}$$

From the DC measurements, we get *C*_*ox*_ ~ 4*fF*/μm^2^. The experimental curve in Fig. 7 uses T_*e*_ as a fitting parameter, getting *T*_*e*_ ~ 320 K in the laser spot region.

### Optoelectronic mixing efficiency

**Fig. 8:**
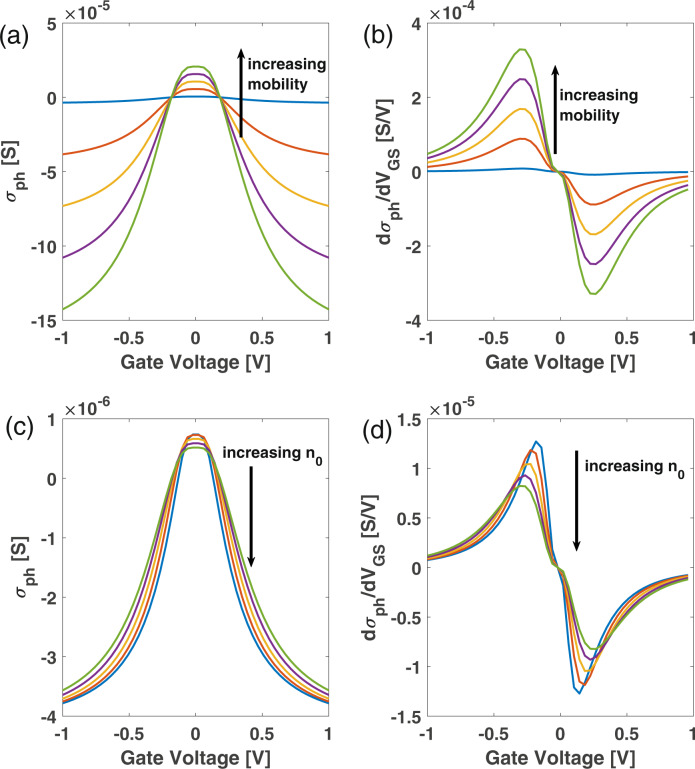
Optoelectronic-mixing performance. **a** Calculated photoconductivity and **b** photoconductivity derivative for *μ*_fe_ varying from 3800 to 100,000 cm^2^ V^−1^ s^−1^. **c** Calculated photoconductivity and **d** photoconductivity derivative for *n*_0_ varying from 10^11^ to 4.5^.^10^11^ cm^−2^.

We now evaluate the effects of *μ* and *n*_0_ on mixing efficiency. Since our SLG is substrate-supported and not encapsulated in hBN, long-range scattering limits conductivity^32^, more specifically Coulomb scattering^32,70,71^. In this regime, the typical *τ* is in the range of hundreds of fs^32^. High-quality SLG can have low *n*_0_ (<10^12^ cm^−2^) and high *μ* > 100,000 cm^2^ V^−1^ s^−1^)^50^. In high-quality SLG encapsulated with hBN, as in ref. ^50^, transport is dominated by random-strain fluctuations^33,72^. For such SLG, a change in *μ* < 20% was measured as a function of *n*^50^. In order to estimate the performance in such SLG, we assume no dependence of *μ* o *n*. Within this assumption, *τ* ∝ *μ* for *μ* >> *K*_*B*_*T*^60^, so Eq. (13) is still valid. For *μ* ~ 100,000 cm^2^ V^−1^ s^−1^, *τ* can be up to 2ps for E_*F*_ ~ 0.2 eV^16^. We thus calculate *σ*_ph_ for *μ* up to 100,000 cm^2^ V^−1^ s^−1^ and *n*_0_ ~ 5 ⋅ 10^11^ cm^−2^, Fig. 8a. By comparing the green and the blue curves, which show the photoconductivity for *μ* ~ 100,000 and 2.500 cm^2^ V^−1^ s^−1^, we predict an increase ~40 times at both low (i.e. *V*_GS_ = 0) and high electrostatic doping (i.e., for *V*_*GS*_ = 1 V). We then differentiate the curves in Fig. 8a. Figure 8b shows an increase in *dσ*_ph_/*dV*_GS_, (i.e. an increase of ${{{\Delta }}}_{{R}_{\rm{ext}}}$) of a factor ~40.

Another parameter that can be improved by employing high-quality SLG is *n*_0_. Fig. 8c plots the photoconductivity for *n*_0_ between 10^11^ cm^−2^ (corresponding to high-quality SLG^50^) and 5 × 10^11^ cm^−2^ (our case), while keeping *μ*_fe_ = 2500 cm^2^ V^−1^ s^−1^. In this case, the photoconductivity increases by a factor ~1.4 near the CNP, as well as at high doping (*V*_*GS*_ = 1 *V*), with *dσ*_ph_/*dV*_GS_ almost doubled, Fig. 8d. This means that the voltage operating point of the device can be decreased.

**Fig. 9:**
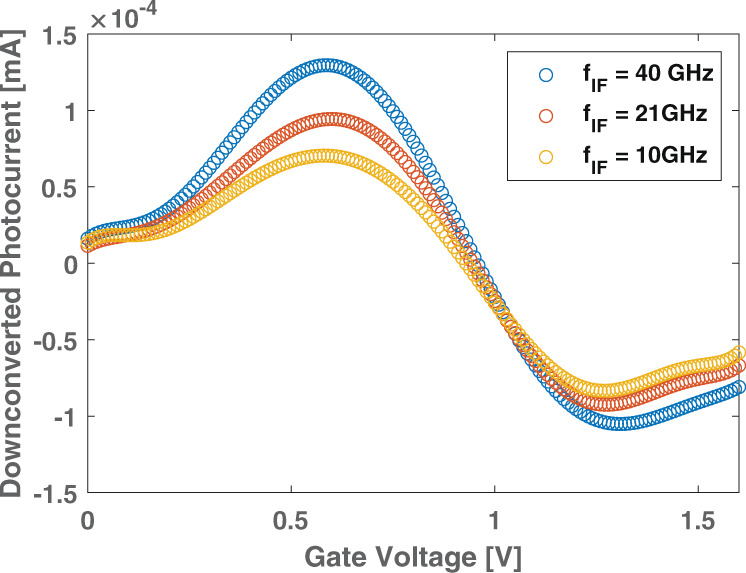
Downconverted photocurrent versus gate voltage at different frequencies. The three curves show the downconverted photocurrent at 10, 21, 40 GHz. The same behavior shown in Fig. 6 is observed, regardless of the operating frequency.

Our prediction is based on the model presented in ref. ^32^, which indicates the screening reduction of the Coulomb impurity scattering mechanism while increasing T^32^. For Coulomb scattering in samples with *μ*_*fe*_ up to 1000 cm^2^ V^−1^ s^−1^^71^, this is supported by several experimental and theoretical works^32,63,67^. For samples with ultra-high *μ*_fe_ up to 100,000 cm^2^ V^−1^ s^−1^^50^), the dominant mechanism limiting *μ*_fe_ is strain disorder^33^, with two contributions: (i) random scalar potential and (ii) random gauge potential^33^. The first is sensitive to T increase owing to screening reduction^33^, similar to what happens for Coulomb scattering^32^, while the second is not^33^. Thus, for ultra-high-quality *μ*_fe_ samples, the “screening reduction” picture may not be accurate. Therefore, we use an intermediate value *μ*_fe_ = 30,000 cm^2^ V^−1^ s^−1^, between 10,000 cm^2^ V^−1^ s^−1^ (i.e., Coulomb scattering regime^71^) and 100,000 cm^2^ V^−1^ s^−1^ (i.e., random-strain fluctuations-induced scattering regime^33^) to infer the performance boost of OEMs based on SLG FET owing to *μ*_fe_ increase. In this case, for *n*_0_ = 10^11^ cm^−2^, we predict an increase of *dσ*_ph_/*dV*_GS_ by a factor ~23 dB. Thus, the downcoversion efficiency in power units can increase ~27dB, overcoming the OEMs state-of-the-art performance^12,31^. Fig. 9 plots the downconverted photocurrent at three frequencies as a function of V_GS_. This shows Fig. 6 is valid regardless of operating frequency.

### Dependence of the laser spot position on photoresponse

**Fig. 10:**
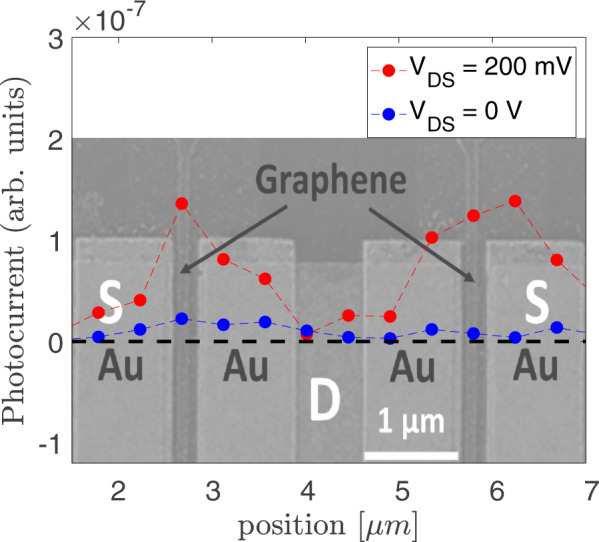
Photoresponse as a function of laser spot position. The red and blue curves show the measured photoresponse as a function of laser spot position along the black dashed cut line, at 200 mV and 0 V, respectively.

Figure 10 shows the photoresponse as a function of laser spot position over the device, along the dashed black line. The photocurrent is measured with the VNA used for the RF measurements in Fig. 2a. The optical beam intensity is modulated at 10 GHz and the optical power impinging the sample is ~10 mW. The beam scan step is ~300 nm, smaller than the spot size of the laser ~2 μm, as defined by the FWHM of the intensity of the Gaussian laser profile. The red dots represent the measured photocurrent at *V*_*DS*_ = 0.2 V, whereas the blue ones are for *V*_DS_ = 0 V. The maximum photocurrent is registered when *V*_DS_ = 0.2 V and the laser spot is over the GFET channel. We observe very low photocurrent, comparable with the instrument noise floor (~−90 dBm electrical power detected over the internal 50Ω impedance of the instrument) at *V*_DS_ = 0. At *V*_DS_ = 0.2 V, we do not observe a change of the photocurrent sign (due to, e.g., asymmetrical heating effect^73^) when the laser spot is scanned from the source to the drain contact. Thus, the role of contacts in the photocurrent generation can be neglected.

## Data Availability

The data that support the findings of this study are available from the corresponding author upon reasonable request.
