# Author Correction: Optoelectronic mixing with high-frequency graphene transistors

**DOI:** 10.1038/s41467-021-23916-0

**Published:** 2021-06-03

**Authors:** A. Montanaro, W. Wei, D. De Fazio, U. Sassi, G. Soavi, P. Aversa, A. C. Ferrari, H. Happy, P. Legagneux, E. Pallecchi

**Affiliations:** 1 Thales Research and Technology, Palaiseau, France; 2 Photonic Networks and Technologies Lab—CNIT, Pisa, Italy; 3 Univ. Lille, CNRS, Centrale Lille, Univ. Polyechnique Hauts-de-France, UMR8520, IEMN Institut d’Electronique de Microélectronique et de Nanotechnologie, Lille, France; 4 Cambridge Graphene Centre, University of Cambridge, Cambridge, UK; 5 LSI, CEA/DRF/lRAMIS, Ecole Polytechnique, CNRS, lnstitut Polytechnique de Paris, Palaiseau, France

**Keywords:** Optics and photonics, Optical properties and devices

Correction to: *Nature Communications* 10.1038/s41467-021-22943-1, published online 12 May 2021.

The original version of this Article contained an error in Fig. 7 and Fig. 8a, c, in which the unit on the vertical axes incorrectly read “μS”. This has been corrected to “S” in both the PDF and HTML versions of the Article.

